# The Prognostic Value of Cardiac Biomarkers in Combination with the SOFA Score for the Evaluation of Sepsis-Related Mortality

**DOI:** 10.3390/medicina62050860

**Published:** 2026-04-30

**Authors:** Vedrana Petrić, Vanja Vlatković, Maria Pete, Dajana Lendak, Siniša Sević, Nadica Kovačević

**Affiliations:** 1 Faculty of Medicine, University of Novi Sad, 21000 Novi Sad, Serbia; maria.pete@mf.uns.ac.rs (M.P.); dajana.lendak@mf.uns.ac.rs (D.L.); sinisa.sevic@mf.uns.ac.rs (S.S.); nadica.kovacevic@mf.uns.ac.rs (N.K.); 2 Clinic for Infectious Disease, University Clinical Center of Vojvodina, 21000 Novi Sad, Serbia; 014855@mf.uns.ac.rs

**Keywords:** sepsis, septic shock, SOFA score, cardiac biomarkers, mortality

## Abstract

*Background and Objectives:* Sepsis is a life-threatening organ dysfunction, and specific biomarkers could improve prognostic assessment in septic patients. The Sequential Organ Failure Assessment (SOFA) score is the standard tool for clinical sepsis monitoring. Recent studies highlight the need for its revision and the identification of rapid, specific, sensitive predictors of sepsis mortality. The aim of this study was to determine the significance of cardiac biomarkers alone or combined with the SOFA score for evaluating sepsis-related mortality. *Materials and Methods:* This is a retrospective, single-center study with a relatively small sample size of 73 septic patients (Sepsis-3 criteria) hospitalized in an intensive care unit (ICU) and intermediate care unit (IMCU). All patients had standard laboratory parameters, cardiac biomarkers, and the SOFA score available upon admission. Statistical analyses included non-parametric Mann–Whitney U test, ROC (Receiver Operating Characteristic) curve analysis, Hanley & McNeil method and Hosmer–Lemeshow goodness-of-fit test. *Results:* Lactate (*p* < 0.001) and SOFA (*p* < 0.001) showed the highest area under the curve (AUC) values, and all cardiac biomarkers had statistically significant AUCs (*p* < 0.05) for sepsis mortality prediction. A comparison of all ROC curves was conducted, but no statistically significant differences were observed. Adding hs-cTn (high-sensitivity cardiac troponin) and lactate to the SOFA score increased its AUC from 0.767 to 0.827 (*p* = 0.421). *Conclusions:* The results showed that non-survivors of sepsis had significantly higher levels of cardiac biomarkers compared to survivors. There were no statistically significant differences in the areas under the ROC curves among the three markers, or between the markers and SOFA. The addition of cardiac biomarkers to SOFA did not improve the discriminatory ability of the SOFA score. Further research with a larger sample size is required to validate and generalize the findings.

## 1. Introduction

Sepsis is a clinical syndrome responsible for high rates of mortality and morbidity worldwide. The new 2016 definition (Sepsis-3) describes sepsis as life-threatening organ dysfunction caused by a dysregulated host response, that is, as an infection accompanied by an increase in the Sequential Organ Failure Assessment (SOFA) score of 2 points or more [1]. Septic shock is accompanied by circulatory, cellular, and metabolic instability, which increases the risk of fatal outcomes. The diagnosis is established when hypotension is present that requires the use of vasopressors to maintain a mean arterial pressure above 65 mmHg, along with a blood lactate level greater than 2 mmol/L after adequate fluid resuscitation [2]. In 2021, the Surviving Sepsis Campaign (SSC) published updated international guidelines for the treatment of sepsis and septic shock. In addition to the new definition, recommendations were provided for clinical monitoring and timely recognition of potentially septic patients through the use of the SOFA (Table A1)*,* quick Sequential Organ Failure Assessment (qSOFA), and Acute Physiology and Chronic Health Evaluation II (APACHE II) scores [3]. Recently published studies have focused on identifying reliable, rapid, easily accessible, and cost-effective biomarkers for mortality prediction in sepsis. Currently, among the 200 known biomarkers of sepsis, none is sufficiently sensitive and specific, which clearly indicates that even the new definition is not entirely adequate [4,5,6,7,8,9].

Septic cardiomyopathy (SCM) is a reversible myocardial dysfunction that occurs in patients with sepsis and septic shock. It results from multifactorial mechanisms. SCM manifests as systolic or diastolic dysfunction of the left or right ventricle, global hypokinesia of the heart, or a localized wall motion abnormality [10,11,12]. Approximately 50% of patients with severe forms of sepsis and septic shock will develop some form of left ventricular systolic dysfunction [12].

The SOFA score is most commonly used for clinical monitoring of sepsis and assessment of organ dysfunction, while the qSOFA score can be used when a rapid evaluation is needed [13]. In recent years, supplementation and revision of the SOFA score have been considered, since new clinical studies and real-world results indicate its shortcomings, particularly regarding the cardiac component. Efforts are being made to incorporate cardiac markers into the SOFA score. The reason why cardiac biomarkers could be combined with the SOFA score lies in their ability to provide an early and accurate picture of cardiac function, even before blood pressure drops or obvious myocardial dysfunction appears [11,14].

Markers that could improve the sensitivity and specificity of the SOFA score include hs-cTn (high-sensitivity cardiac troponin), N-terminal pro-brain natriuretic peptide (NT-proBNP), Creatine Kinase–MB (CK-MB), and lactate [11,15,16]. Incorporating these parameters into the existing SOFA score may enhance risk stratification and improve the identification of patients at high risk of mortality. This would allow timely initiation of adequate therapy, thereby reducing complications and improving outcomes [11].

Beyond sepsis, cardiac biomarkers may be elevated in a variety of other clinical conditions such as acute coronary syndromes, acute and chronic heart failure, pulmonary embolism, myocarditis, tachyarrhythmias, and hypertensive crises. Elevated concentrations are also reported in patients with chronic kidney disease due to reduced clearance. Therefore, while these biomarkers may have prognostic value in sepsis, their elevation is not disease-specific and should be interpreted within the overall clinical context [17].

The aim of this exploratory study was to determine the significance of cardiac biomarkers alone and in combination with the SOFA score for the evaluation of sepsis-related mortality.

## 2. Materials and Methods

### 2.1. Study Design

This is a retrospective, exploratory, single-center study with a relatively small sample size of 73 septic patients (Sepsis-3 criteria) hospitalized in an intensive care unit (ICU) and intermediate care unit (IMCU).

Septic patients were identified according to the Sepsis-3 criteria. Between 1 January 2024, and 31 December 2024, a total of 160 septic patients were hospitalized at the Clinic for Infectious Diseases University Clinical Center of Vojvodina (UKCV) and were included in this single-center retrospective study, which was approved by the Ethics Committee of UKCV (approval number 00-132).

Data were collected retrospectively from electronic healthcare records for all consecutive patients diagnosed with sepsis; consequently, additional informed consent was not required.

### 2.2. Patient Selection

The exclusion criteria (clinical criteria and no ICU/IMCU admission criteria) were designed to minimize confounding factors and to create a more homogenous sample. Clinical exclusion criteria: patients younger than 18 years, patients with pre-existing cardiovascular conditions, respiratory, renal and immunological conditions and others. Additionally, patients who did not need intensive care unit (ICU) admission or intermediate care admission (IMCU) were excluded. Consequently, in our hospital, biomarker testing was not routinely performed in all patients, although cardiac markers were routinely measured in all patients admitted to the ICU and IMCU, and may have preferentially included more severely ill patients.

**Figure 1:**
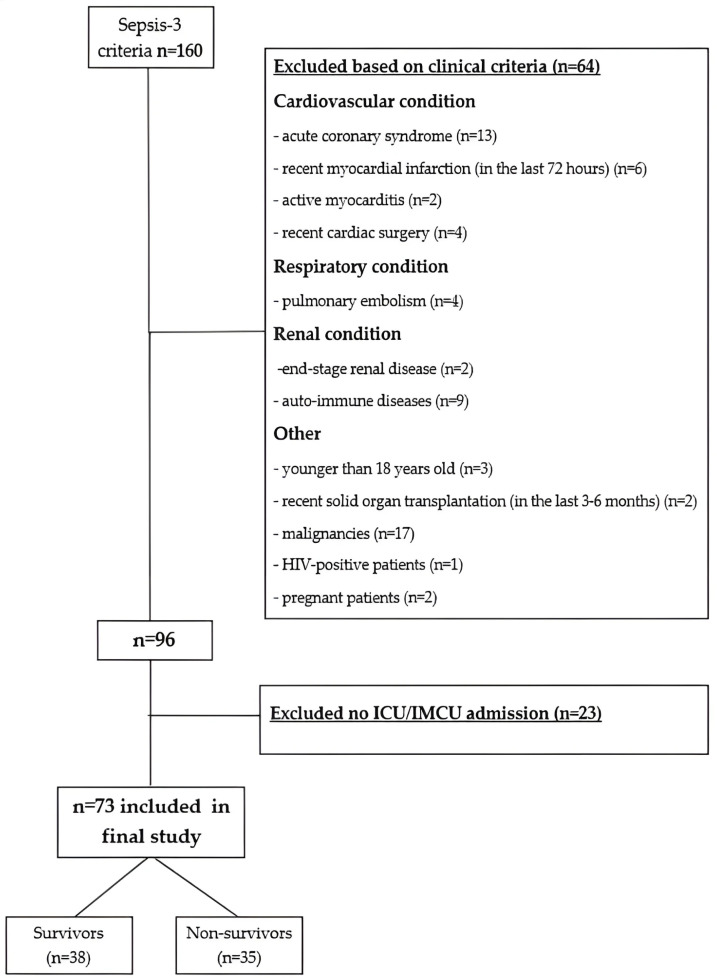
Patient selection flowchart.

No patients were excluded due to missing data. A total of 73 patients were included in the final analysis and were divided into two groups: survivors and non-survivors (Figure 1).

These selections and the final cohort may not fully reflect the general sepsis population, introducing potential selections bias. While this reflects real-world clinical practice, it may limit the generalizability of the findings; however, the statistical associations were analyzed only within a clearly defined cohort with complete data, thereby preserving the internal validity of the predictive model.

### 2.3. Laboratory Testing

For all patients included in the study, demographic data, clinical characteristics, vital signs at admission, comorbidities, length of stay, source and cause of infection and outcomes were recorded. All patients had following routine laboratory analyses at admission: complete blood count (CBC) with white blood cell count (WBC), neutrophil (Neu), lymphocyte cell count (Limf)), sodium (Na), potassium (K), magnesium (Mg), concentrations of bilirubin; C-reactive protein (CRP), procalcitonin (PCT) and D-dimer, alanine aminotransferase (ALT), aspartate aminotransferase (AST), and gamma-glutamyl transferase (GGT).

All patients had serum cardiac biomarkers (hs-cTn, NT-proBNP, and CK-MB), blood gas analysis (lactate, pH (potential of hydrogen), pO_2_ (partial pressure of oxygen), pCO_2_ (partial pressure of carbon dioxide), bicarbonate and base excess). The neutrophile to lymphocyte ratio (NLR) was calculated by dividing the absolute neutrophil count by the absolute lymphocyte count. Mean arterial pressure (MAP) was calculated from measured systolic and diastolic blood pressure values. The SOFA score, qSOFA score and Glasgow Coma Scale (GCS) were recorded.

Routine parameters derived from blood were determined using the CELL-DYN Sapphire ABBOT machine (Abbot Park, IL, USA), via flow cytometry with commercially available kits from the same vendor. CRP concentrations were determined using an immunoturbidimetric assay (ABX Micros, Horiba ABX SAS, Montpellier, France) and expressed in mg/L. PCT concentrations were determined using an automatic analyser (mini Vidas, bioMérieux, Marcy-l’Étoile, France), with a detection limit of 0.05 ng/L.

Cardiac biomarkers were measured using the VIDAS System (bioMérieux, Marcy-l’Étoile, France), an automated immunoassay platform based on enzyme-linked fluorescent assay (ELFA) technology. Venous blood samples were collected, centrifuged, and serum was separated for immediate analysis. The concentrations of hs-cTn, CK-MB, NT-proBNP were determined according to the manufacturer’s instructions. Results were expressed in ng/mL.

Blood gas analysis was performed on a Gem Premier 3500 analyzer (Instrumentation Laboratory, Bedford, MA, USA). The device, utilizing Intelligent Quality Management (iQM) technology, was used for the real-time measurement of (pH, pCO_2_, pO_2_, bicarbonate, base excess and lactate), offering automatic quality assurance with each sample.

### 2.4. Statistical Analysis

Statistical analyses were performed using IBM SPSS Statistics, version 21.0 (IBM Corp., Armonk, NY, USA). Data with a normal distribution are presented as mean ± standard deviation ($\bar{X}$ ± SD), data with a skewed distribution are presented as median with interquartile range (IQR). Categorical variables were presented numerically and with percentages (N(%)), and comparisons were analyzed using the Chi-square test (χ^2^ test). For numerical data, the t-test was used to compare groups when the data followed a normal distribution, and the Mann–Whitney U test was performed for non-normally distributed data.

For all individual biomarkers (hs-cTn, CK-MB, NT-proBNP), the SOFA score, and hematological and biochemical parameters (red blood cell (RBC) count, red cell distribution width (RDW), neutrophils (Neu), lymphocyte cell count (Limf), neutrophil-to-lymphocyte ratio (NLR), platelets (PLT), hemoglobin (Hb), CRP, and lactate), we constructed Receiver Operating Characteristic (ROC) curves and calculated the areas under the curve (AUCs), optimal cut-off values, sensitivity, and specificity.

Univariate analysis was performed to evaluate the predictive value of individual cardiac biomarkers for mortality. Due to the limited sample size, multivariate analysis was not performed, as it would not provide statistically reliable estimates. The independent prognostic value of the biomarkers could not be established.

The predictive performance of the combined models incorporating the SOFA score and selected cardiospecific biomarkers was assessed using ROC analysis. The biomarkers included in the combined models were those demonstrating the highest area under the curve (AUC) values in the individual ROC analyses. As lactate demonstrated the highest area under the curve (AUC) value, followed by the SOFA score and hs-cTn, a modified SOFA score was constructed by first incorporating hs-cTn and subsequently both hs-cTn and lactate. For hs-cTn and lactate, values within the reference range were assigned a score of 0, whereas values above the reference range were divided into quartiles and assigned scores from 1 to 4, consistent with the scoring structure of the original SOFA components.

The modified scores were then calculated by summing the SOFA and troponin points, and a corresponding ROC curve was generated. Subsequently, a combined score including the SOFA score, hs-cTn, and lactate was constructed, and its ROC curve was analyzed. Calibration of the model was evaluated using the Hosmer–Lemeshow goodness-of-fit test, with a *p*-value > 0.05 indicating good agreement between predicted and observed outcomes. This model needs internal or external validation.

The statistical significance of the difference between two ROC curves was assessed using the Hanley & McNeil method implemented in MedCalc software version 23.2.0 (MedCalc Software Ltd, Ostend, Belgium).

A *p*-value < 0.05 was considered statistically significant. No correction for multiple comparisons was applied because the analyses were primarily exploratory based on clinical interest. Applying such corrections could increase the risk of type II error and reduce statistical power. The results are presented in tables and figures with textual interpretation.

## 3. Results

Out of the 73 included patients in this study 37/73 (50.7%) were females and 36/73 (49.3%) were males (Table 1).

Average age was 72.7 ± 11.14 years (range: 41–93), indicating a predominance of the geriatric population.

The overall mortality was 35/73 (47.9%) (Table 1).

There was no statistically significant difference between the two groups in terms of age, gender, comorbidities, site of infection, source of infection and length of stay (Table 1.).

Significantly higher values of respiratory rate (RR) was observed in the non-survivor group, and other vital signs did not differ significantly between two groups. MAP was significantly lower in non-survivor group (Table 1).

There was a statistically significant difference in SOFA and qSOFA scores, and both scores were higher in non-survivors (*p* < 0.001). However, GCS also differed significantly between the groups (*p* < 0.001) (Table 1).

Among all patients with sepsis in ICU and IMCU, 20 (27.39%) developed septic shock. There was a significant difference between the groups of patients with septic shock (*p* < 0.001) (Table 1).

Comparing the levels of all cardiac biomarkers between the groups, significantly higher values were observed in non-survivors group (Table 2).

There was a statistically significant difference in magnesium levels (*p* = 0.049), AST (*p* = 0.006), GGT (*p* < 0.001) and D dimer (*p* < 0.001). There was no significant difference in other routine laboratory parameters (Table 2).

Non-survivors had statistically significantly higher lactate levels (*p* < 0.001), as well as statistically significantly lower values of pCO_2_, bicarbonate, and base excess (*p* = 0.04, *p* = 0.048, and *p* = 0.01, respectively) (Table 2).

**Figure 2:**
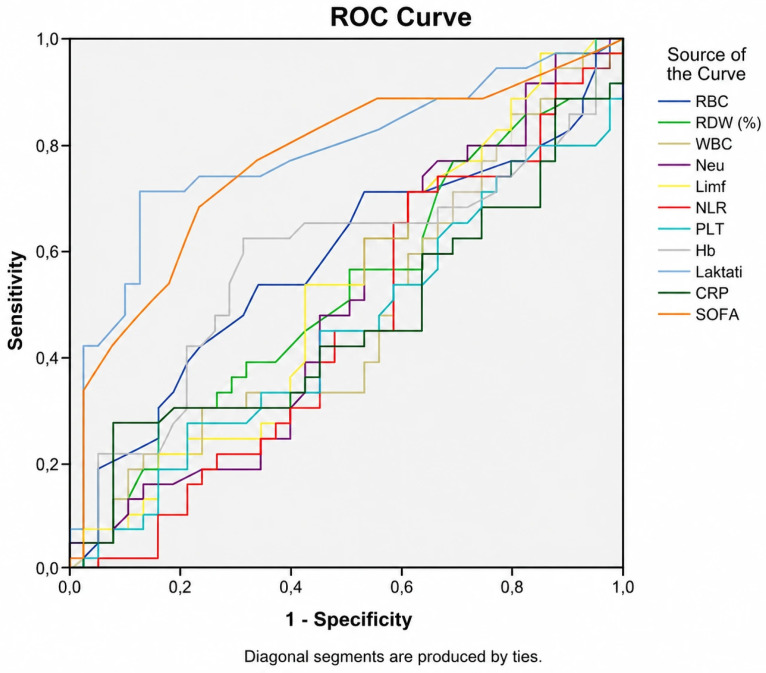
ROC curves for RBC, RDW, WBC, Neu, Limf, NLR, PLT, CRP, Hb, and lactate.

The ROC curves analysis demonstrated that lactate and SOFA score had the greatest area under the curve (AUC) for predicting sepsis mortality. AUC, ROC, cut-off, sensitivity and specificity are shown in Table 3 and Figure 2.

**Figure 3:**
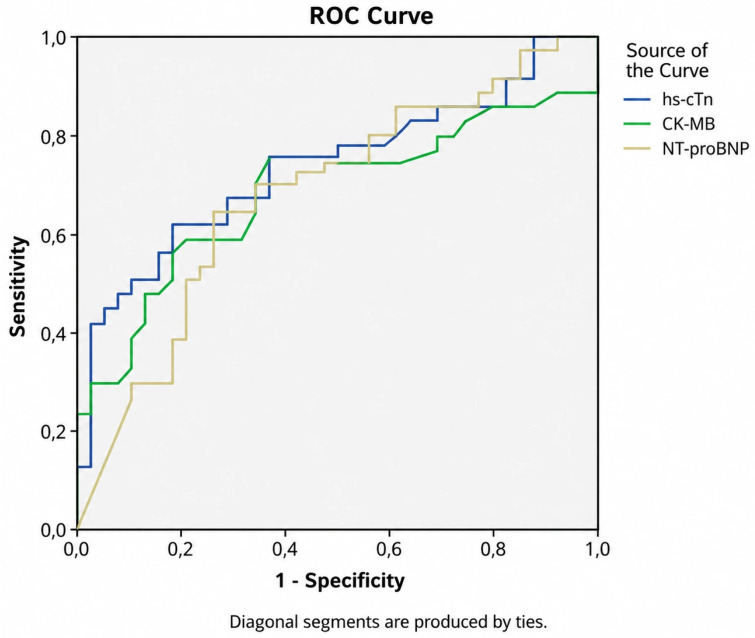
ROC curves for cardiac markers.

ROC curve analysis demonstrated that hs-cTn had the highest discriminative ability for predicting mortality, as reflected by the highest AUC. AUC, ROC, cut-off, sensitivity and specificity are shown in Table 4 and Figure 3.

In order to assess whether there is a statistically significant difference in discriminative performance between cardiac biomarkers, SOFA score and lactate, we used comparation of ROC curves (Hanley & McNeil method). However, none of the pairwise comparisons revealed statistically significant differences between AUC values. These findings indicate that although each parameter showed significant individual discriminatory ability, their predictive performance was not statistically distinguishable from one another within the present cohort (Table 5).

Univariate analysis demonstrated that higher concentrations of all cardiac biomarkers and the SOFA score indicated higher risk of mortality. Due to the highly skewed distribution of some biomarkers (hs-cTn and NT-proBNP), log-transformation was applied prior to analysis to obtain more appropriate and interpretable effect estimates (Table 6).

For the identification of independent predictors of sepsis mortality, multivariable regression analysis was required. Multivariable regression analysis was not conducted because of the limited sample size and small number of events, which would violate the underlying statistical assumptions, particularly with respect to the events-per-variable (EPV) rule and model stability. Therefore, we emphasize that statistical significance was observed primarily in the univariate analysis and the findings should be interpreted as exploratory.

**Figure 4:**
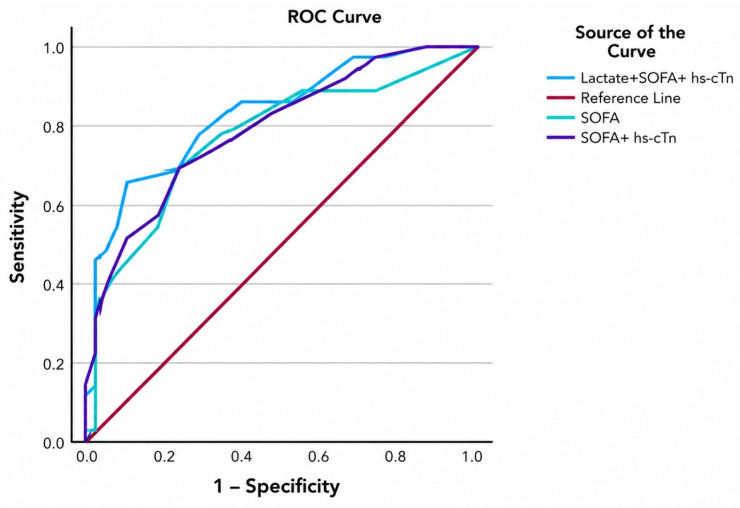
ROC curves for combinations of cardiac biomarkers.

Given the limited sample size and the inability to perform a multivariable regression analysis, the predictive performance of different combinations of the SOFA score and cardiac biomarkers were evaluated using ROC curve analysis (Figure 4).

ROC analysis of biomarker combinations for the prediction of mortality did not reach statistical significance. However, the model showed that adding hs-cTn to the SOFA score increased the ROC AUC from 0.767 to 0.789, although the difference between the two AUC values was not statistically significant (*p* = 0.775) (Table 7).

Further addition of lactate to the modified score that already included both the SOFA score and hs-cTn increased the ROC AUC from 0.767 to 0.827; however, this difference also did not reach statistical significance (*p* = 0.421).

## 4. Discussion

The pathophysiological mechanisms of sepsis-induced cardiovascular dysfunction are still not entirely understood, and the presence of cardiovascular dysfunction is associated with a significantly increased mortality rate [11,18], as confirmed by the results of our study (47.9% of the study participants), but the high incidence of mortality observed in our study is inconsistent with the findings from a recent study [7,19]. A potential explanation for this difference may lie in the fact that our study included only patients hospitalized in the ICU and IMCU, representing a population with more severe forms of sepsis. Consequently, the resulting potential selection bias may limit the generalizability of our findings.

According to these data, the timely identification and implementation of potentially highly specific, rapid, useful, low-cost, available, and sensitive biomarkers for predicting mortality in septic patients is of great importance [19]. Many studies have investigated the importance of various biomarkers that are more or less specific to sepsis, as well as combinations of biomarkers, for the early recognition of sepsis severity and their potential role in predicting mortality [20,21]. Therefore, our primary aim was to examine the ability of cardiac biomarkers and the SOFA score, alone or in combination, for the evaluation of sepsis-related mortality in routine clinical practice in middle-income countries.

The results of our study show a predominance of older patients, with an almost equal distribution of males and females. Similar age-related findings have been reported by other authors [11,22]. Almost half of our patients had three or more chronic diseases.

In a previous study conducted in China, the most common comorbidities were hypertension (40.1%), diabetes (21.9%), cerebrovascular diseases (17.2%), and COPD (12.2%), which aligns with the data observed in our study and in other recent studies [11,23].

Vincent JL et al., in their retrospective study, reported that pneumonia was the primary cause of sepsis, with the respiratory tract being the most common site of infection, and the urinary tract was the second most common site [19,24]. Our data show that 41.09% of patients had a urinary tract infection. A possible explanation for these results is the presence of a specialized institution for treating respiratory tract infections in Vojvodina, namely the Institute for Pulmonary Diseases in Sremska Kamenica. The percentage of undetermined sources of infection in our study was significantly higher (26.3%) than in previous studies, exceeding the global average, which ranges between 6% and 21% [25].

A comparison of routinely tested laboratory parameters, inflammatory biomarkers, cardiac markers between survivors and non-survivors showed that all cardiac biomarkers, Mg, AST, D-dimer and lactate had significantly higher values in non-survivors than in survivors. These results indicate multisystem derangements, including increased oxidative stress, inflammatory response, and ischemia–reperfusion injury [11,14,26,27,28,29,30]. These findings from our study show that all cardiac biomarkers were significantly elevated in the non-survivor group, suggesting a role in sepsis prognosis and informing the approach to critically ill septic patients with elevated cardiac biomarkers.

The results of our study show that lactate and the SOFA score had better predictive value for sepsis mortality, with higher AUC ROC then cardiac biomarkers. This could be explained by the fact that both SOFA score and lactate are primarily used to assess organ failure.

In an Australian study, lactate was identified as the strongest predictor of mortality in multivariate analysis [31].

Several studies have shown that elevated lactate levels in sepsis are associated with mortality [32,33].

But there are currently controversies regarding the use of the SOFA score in clinical practice. A 2023 study by Moreno et al. suggested that the SOFA score should be updated [34]. Since new clinical studies and real-world results indicate its shortcomings, particularly regarding the cardiac component, efforts are being made to incorporate cardiac biomarkers into the SOFA score. The reason why cardiac biomarkers could be combined with the SOFA score lies in their ability to provide an early and accurate picture of cardiac function, even before blood pressure drops or obvious myocardial dysfunction appears [11,14,28]. Lee et al. developed a modified CV SOFA score (cardiovascular SOFA), due to changes in vasopressor use. This study was based on data from multiple cohorts. Even though this score was not for global use because of multiple limitations, it could be a good starting point for SOFA score modification and other new research [28]. A 2024 study developed a cardiac-extended SOFA score model by adding hs-cTnT, NT-proBNP, heart rate, and presence of atrial fibrillation. Even this model did not significantly improve the discriminatory ability for 30-day mortality compared to the standard SOFA score; therefore, future studies are needed to investigate this model and the revised cardiovascular component of the SOFA score [29].

Results from our study show that among the cardiac biomarkers, hs-cTn had the highest AUC; however, there was no statistically significant difference between the areas under the ROC curves of the three markers. Although hs-cTn showed higher AUC values in certain cohorts, these findings indicate that it does not consistently demonstrate superior discriminatory performance compared with other cardiac biomarkers for outcome prediction in sepsis when evaluated using ROC-based analyses. It is well known that elevated troponin levels in sepsis are associated with sepsis induced myocardial injury [30,35]. Recent meta-analyses suggest a significant association between troponin elevation and increased mortality in patients with sepsis, emphasizing the need for a better understanding of this relationship [35].

The findings also showed a significant association between cardiac troponin elevation and mortality among septic patients [35].

Univariate analysis was performed to evaluate the association between the SOFA score, individual biomarkers, and sepsis mortality. In this analysis, all three cardiac markers demonstrated statistical significance; however, these findings should be considered only exploratory. To establish an independent prognosticator of mortality, multivariate analysis using binary logistic regression would be needed.

Because of the limited sample size and the inability to perform multivariable regression analysis, we evaluated the discriminatory performance of different combinations of the SOFA score, serum lactate, and cardiospecific biomarkers using ROC curve analysis. Therefore, this limits direct comparability with previously published studies.

This model was a practical choice intended to provide clinically interpretable stratification in the absence of validated cut-off values for cardiac biomarkers. This approach requires both internal and external validation before clinical implementations. Interpretation of this analysis should be considered preliminary.

The calibration of both models was evaluated using the Hosmer–Lemeshow goodness-of-fit test.

This study further found, through ROC curve analysis, that the combination of the SOFA score and cardiac markers for predicting mortality had a higher AUC than the SOFA score alone, but the difference did not reach statistical significance.

Similarly, the AUC of the combined model integrating the SOFA score, hs-cTn and lactate was superior to that of both the SOFA score alone and the SOFA score plus hs-cTn model; however, no statistically significant differences were observed.

One study showed that the combination of different biomarkers had better prognostic value than SOFA score alone in predicting sepsis mortality [36]. Our results, however, may have been affected by selection bias and the small sample size. Due to methodological and statistical limitations, we were unable to identify a statistically significant prognostic combination.

Many studies have attempted to modify the cardiovascular component of the SOFA score to better reflect cardiac dysfunction and improve its predictive performance (11, 14, 16). Accordingly, the present study should be interpreted as exploratory, with the main focus on assessing model discrimination (ROC/AUC) rather than developing a predictive model. The development of a predictive model would require a larger sample size as well as multivariable regression analysis.

Our study has several limitations. First, to the best of our knowledge, this is the first study in our clinical setting exploring the association between cardiac biomarkers and sepsis mortality. Second, this is a single-center study, including only patients hospitalized in ICU and IMCUs (critically ill patients), and cardiac biomarkers were not measured systematically in all septic patients. This approach and this select cohort limit the generalizability of our findings to other populations. Therefore, the study results should be interpreted strictly as exploratory. Third, although we adjusted for as many covariates as possible to diminish their possible influences, owing to the retrospective design of the study, residual confounding may still exist, which needs to be investigated in the future. Fourth, the relatively small number of patients represents an additional limitation for proper statistical analysis (multivariate analysis); the lack of correction for multiple testing may have increased the risk of false positive results and the results should be validated in larger studies. The modified SOFA-based score should be interpreted as an exploratory tool, and needs internal or external validation before clinical implementation. Interpretation of this model should be considered preliminary. Finally, the primary aim of this study was exploratory, focusing on the assessment of discriminatory performance (ROC/AUC) rather than the development of a fully validated predictive model.

## 5. Conclusions

Based on our findings, cardiac biomarkers were significantly higher in non-survivors compared to survivors. There was no statistically significant difference between the areas under the ROC curves of the three markers, neither between them nor SOFA.

The addition of cardiac biomarkers to SOFA did not improve the discriminatory ability of the SOFA score. Further research with a larger sample size is required to validate and generalize the findings.

Due to the complex pathophysiology of sepsis, combined biomarkers may represent the future of predicting outcomes among patients.

## Figures and Tables

**Table 1:** Demographic characteristics of the sample, comparation of comorbidity structure, sites of infection, isolated pathogens, hemodynamic parameters between survivors and non-survivors.

VARIABLE	Survivors (N = 38)	Non-Survivors (N = 35)	*p*
Age (Mean ± SD)	71.50 ± 12.77	73.00 ± 9.20	0.624 **
Gender N(%)			
Male	19 (50.0%)	17 (48.6%)	0.903 *
Female	19 (50.0%)	18 (51.4%)	
Comorbidity structure N(%)			
Arterial hypertension	27 (71.1%)	29 (82.9%)	0.233 *
Diabetes mellitus	13 (35.1%)	18 (51.4%)	0.163 *
Chronic obstructive pulmonary disease (COPD)	4 (10.5%)	3 (8.6%)	0.777 *
Asthma	2 (5.3%)	2 (5.7%)	0.933 *
Sepsis source N(%)			
Lungs	2 (5.26%)	3 (8.57%)	0.4927 *
Central nervous system infection (CNS)	2 (5.26%)	5 (14.28%)	
Abdomen	4 (10.52%)	3 (8.57%)	
Skin	2 (5.26%)	4 (11.42%)	
Not identified	10 (26.32%)	8 (22.86%)	
Microbiologically confirmed bacteria from blood culture N(%)	14 (36.8%)	14 (40.0%)	****
*Escherichia coli* (*E. coli*)	8 (21.1%)	5 (14.3%)	
*Klebsiella pneumoniae* (*K. pneumoniae*)	2 (5.3%)	2 (5.7%)	
*Pseudomonas aeruginosa* (*P. aeruginosa*)	1 (2.6%)	1 (2.9)	
*Acinetobacter baumanii*	0 (0.0%)	1 (2.9%)	
*Vancomycin resistant enterococcus*	1 (2.6%)	0 (0.0%)	
Other	2 (2.6%)	5 (14.3%)	
Vital Signs			
Heart rate (beats per minute—bpm); (Mean ± SD)	95.00 ± 21.35	87.00 ± 26.23	0.728 **
Respiratory rate (RR) (Median (IQR))	15 (13–16.5)	18 (15–21)	0.003 ***
Body temperature (Median (IQR))	37.00 (36.6–38.7)	37.00 (36.7–38.2)	0.214 ***
Oxygen saturation (SaO_2_) (Median (IQR))	96 (94–98)	95.2 (93–98)	0.398 ***
MAP (Mean ± SD)	86.67 ± 18.85	73.33 ± 21.54	0.037
SOFA (Median (IQR))	4 (4–5)	7 (7–9)	<0.001
qSOFA (Median (IQR))	0 (0–1)	1 (1–2)	<0.001
GCS (Median (IQR))	15 (10–15)	9 (6–15)	<0.001
Septic shock N(%)	3 (7.9%)	17 (48.6%)	<0.001
Length of stay (Median (IQR))	12 (10.5–32.5)	2 (2–19)	0.051

χ^2^ test *; *t* test **; Mann–Whitney U test ***; *p* value is not calculated due to small numbers in groups ****.

**Table 2:** Differences in standard laboratory parameters, blood gas analysis and cardiac biomarkers between survivors and non-survivors.

VARIABLE	Survivors (N = 38)	Non-Survivors (N = 35)	*p*
WBC ^†^ [×10^9^/L]	16.71 (10.6–20.2)	14.87 (10.8–20.9)	0.947
Neu ^†^ [%]	88.90 (81.3–91.7)	89.10 (83.3–91.1)	0.847
Limf ^†^ [%]	5.60 (3.5–11.1)	6.20 (4.1–12.0)	0.600
NLR ^†^	16.29 (7.38–25.87)	14.15 (6.94–21.78)	0.612
PLT ^†^ [×10^9^/L]	208.00 (141.75–291.0)	183.00 (128.0–296.0)	0.547
RBC [×10^12^/L]	4.09 ± 0.81 *	4.32 ± 0.91	0.255 *
RDW ^†^ [%]	14.40 (13.3–15.97)	14.60 (13.6–16.3)	0.607
Hb [g/L]	121.63 ± 24.98	129.97 ± 31.03	0.208 *
Hct ^†^ [L/L]	0.35 (0.32–0.40)	0.40 (0.31–0.44)	0.198
CRP [mg/L]	227.08 ± 103.40	221.51 ± 132.04	0.841 *
PCT ^†^ [ng/mL]	9.20 (3.54–40.04)	14.60 (2.30–79.72)	0.787
Fibrinogen ^†^ [g/L]	5.46 (4.32–8.55)	4.64 (3.48–8.13)	0.307
Na ^†^ [mmol/L]	137.00 (134.0–141.0)	139.00 (135.0–143.0)	0.226
K ^†^ [mmol/L]	3.90 (3.5–4.2)	4.30 (3.4–4.8)	0.114
Mg ^†^ [mmol/L]	0.81 (0.66–0.90)	0.89 (0.74–0.96)	0.049
Urea ^†^ [mmol/L]	14.15 (7.85–22.07)	17.50 (9.90–26.30)	0.140
ALT ^†^ [U/L]	29.50 (20.5–44.2)	40.00 (23.0–109.0)	0.139
AST ^†^ [U/L]	30.00 (20.75–62.75)	59.00 (40.0–141.0)	0.006
GGT ^†^ [U/L]	28.50 (19.0–65.75)	51.00 (27.0–113.0)	0.022
D dimer ^†^ [mg/L] FEU]	2.26 (1.5–3.7)	4.38 (3.5–12.7)	<0.001
Hs-cTn ^†^ [ng/L]	27.00 (12.6–133.29)	172.20 (27.5–1676.9)	0.001
CK-MB ^†^ [U/L]	21.00 (13.0–33.0)	36.00 (17.0–79.0)	0.009
NT-pro BNP ^†^ [pg/mL]	2603.50 (1526.5–4971.0)	5024.00 (2516.0–25,000.0)	0.009
Laktati ^†^ [mmol/L]	1.40 (1.07–1.82)	2.70 (1.60–4.60)	<0.001
pH ^†^	7.42 (7.35–7.44)	7.38 (7.27–7.45)	0.246
_P_O_2_ ^†^ [mmHg]	75.00 (66.5–88.0)	79.00 (65.0–103.0)	0.154
_P_CO_2_ ^†^ [mmHg]	35.00 (32.0–38.0)	33.00 (26.0–36.0)	0.040
Bicarbonate ^†^ [mmol/L]	21.95 (20.07–25.60)	20.10 (12.60–23.30)	0.048
Base excess ^†^ [mmol/L]	−0.95 (−4.0–0.90)	−5.00 (−9.7–−0.5)	0.010

Mean ± SD; Median (IQR) ^†^; Mann–Whitney U test *.

**Table 3:** Diagnostic value of laboratory parameters and SOFA score in predicting mortality in sepsis.

Variables	AUC (95% Confidence Interval (CI *))	*p*	Cut-Off Value	Sensitivity (%)	Specificity (%)
RBC [×10^12^/L]	0.588 (0.454–0.721)	0.189	4.2	54.3	65.8
RDW [%]	0.535 (0.401–0.669)	0.608	16.1	31.4	81.6
WBC [×10^9^/L]	0.495 (0.361–0.630)	0.947	16.54	65.7	52.6
Neu [%]	0.513 (0.379–0.648)	0.847	91.2	20.0	65.8
Limf [%]	0.536 (0.402–0.669)	0.600	2.8	97.1	15.8
NLR	0.465 (0.332–0.599)	0.612	18.69	68.6	44.7
PLT [10^9^/L]	0.459 (0.325–0.593)	0.547	104	20.0	94.7
Hb [g/L]	0.585 (0.449–0.721)	0.212	126	62.9	68.4
Lactate[mmol/L]	0.785 (0.676–0.895)	<0.001	2.1	71.4	86.8
CRP [mg/L]	0.475 (0.338–0.612)	0.711	333	71.4	7.9
SOFA	0.767 (0.655–0.879)	<0.001	5	68.6	76.3

CI—Confidence interval *.

**Table 4:** Diagnostic value of cardiac biomarkers in predicting sepsis mortality.

Variables	AUC (95% CI)	*p*	Cut-Off Value	Sensitivity (%)	Specificity (%)
hs-cTn [ng/L]	0.729 (0.610–0.847)	0.001	146.200	60.0	81.6
CK-MB [U/L]	0.679 (0.550–0.808)	0.009	26.50	74.3	63.2
NT-proBNP [pg/mL]	0.677 (0.553–0.801)	0.009	4385.00	62.90	73.7

**Table 5:** Statistical comparison of ROC curves (Hanley & McNeil method).

Comparation Between ROC Curves	*p*	Standard Error	Difference	Z Statistic
Lactate vs. SOFA	0.822	0.079	0.018	0.225
Lactate vs. hs-cTn	0.495	0.082	0.056	0.682
Lactate vs. CK-MB	0.221	0.087	0.106	1.225
Lactate vs. NT-proBNP	0.200	0.084	0.108	1.281
SOFA vs. hs-cTn	0.646	0.083	0.038	0.459
SOFA vs. CK-MB	0.313	0.087	0.088	1.009
SOFA vs. NT-proBNP	0.289	0.085	0.090	1.059
hs-cTn vs. CK-MB	0.575	0.089	0.050	0.561
hs-cTn vs. NT-proBNP	0.550	0.087	0.052	0.598
CK-MB vs. NT-proBNP	0.982	0.089	0.002	0.022

**Table 6:** Analysis of the individual impact of biomarkers and the SOFA score on mortality in patients with sepsis—results of univariate analysis.

Variables	Univariate			
	*p*	Odds Ratio (OR)	95% CI	
			Lower Limit	Upper Limit
hs-cTn (log10) [ng/L]	0.001	2.766	1.518	5.041
CK-MB [U/L]	0.030	1.020	1.002	1.039
NT-Pro BNP (log10) [pg/mL]	0.015	2.937	1.237	6.976
SOFA	<0.001	1.450	1.180	1.781

**Table 7:** ROC analysis of biomarker combinations for prediction of mortality.

Test Variable(s)	AUC (95% CI)	Standard Error	*p*	Cut-Off	Sensitivity (%)	Specificity (%)
Lactate+ SOFA+ hs-cTn	0.827 (0.732–0.922)	0.048	<0.001	8	77	81
SOFA+ hs-cTn	0.789 (0.686–0.892)	0.052	<0.001	7	74	66
SOFA	0.767 (0.655–0.879)	0.057		5	77	66

The calibration of both models was evaluated using the Hosmer–Lemeshow goodness-of-fit test. The SOFA+ hs-cTn model demonstrated a *p* value of 0.908, whereas the Lactate+ SOFA+ hs-cTn model had a *p* value of 0.327, suggesting good agreement between predicted and observed outcomes for both models.

## Data Availability

The data presented in this study are available from the first author upon request.

## Abbreviations

ALT	Alanine aminotransferase
APACHE II	Acute Physiology and Chronic Health Evaluation II
AST	Aspartate aminotransferase
AUC	Area under the curve
CI	Confidence interval
CK-MB	Creatine Kinase–MB
CNS	Central nervous system
COPD	Chronic obstructive pulmonary disease
CRP	C-reactive protein
ELFA	Enzyme-linked fluorescent assay
EPV	Events-per-variable
GCS	Glasgow Coma Scale
GGT	Gamma-glutamyl transferase
Hb	Hemoglobin
HCT	Hematocrit
HIV	Human immunodeficiency virus
hs-cTn	High-sensitivity cardiac troponin
ICU	Intensive care unit
IMCU	Intermediate care unit
IQR	Interquartile range
IQM	Intelligent Quality Management
K	Potassium
Limf	Lymphocyte cell count
MAP	Mean arterial pressure
Mg	Magnesium
N	Number of observed parameters
Na	Sodium
Neu	Neutrophil
NLR	Neutrophil to lymphocyte ratio
NT-proBNP	N-terminal pro-brain natriuretic peptide
OR	Odds ratio
pCO_2_	Partial pressure of carbon dioxide
PCT	Procalcitonin
pH	Potential of hydrogen
PLT	Platelets
pO_2_	Partial pressure of oxygen
qSOFA	Quick Sequential Organ Failure Assessment
RBC	Red blood cell count
RDW	Red cell distribution width
ROC	Receiver Operating Characteristic
RR	Respiratory rate
SaO_2_	Oxygen saturation
SCM	Septic cardiomyopathy
SD	Standard deviation
SOFA	Sequential Organ Failure Assessment
SSC	Surviving Sepsis Campaign
UKCV	University Clinical Center of Vojvodina
WBC	White blood cell count
χ^2^ test	Chi-square test

## Appendix A

**Table A1:** Parameters for calculating the SOFA score.

System	Parameter	0	1	2	3	4
Respiratory	PaO_2_/FiO_2_ (mmHg)	≥400	<400	<300	<200 with mechanical ventilation	<100 with mechanical ventilation
Coagulation	Platelets (10^9^/L)	≥150	<150	<100	<50	<20
Liver	Bilirubin (mg/dL)	<1.2	1.2–1.9	2–5.9	5–11.9	>12
Cardiovascular	MAP or need for vasopressors	>70	<70	Dopamine < 5; ILI dobutamine	Dopamine 5, 1–15; ILI epinephrine < 0.1; ILI norepinephrine < 0.1	Dopamine >15; ILI epinephrine > 0.1; ILI norepinephrine > 0.1
CNS	Glasgow Coma Scale (GCS)	15	13–14	10–12	6–9	<6
Kidneys	Creatinine (mg/dL) or urine output	<1.2	1.2–1.9	2–3.4	3.5–4.9	>5
